# Chiral Zn(II)-Bisamidine Complex as a Lewis-Brønsted Combined Acid Catalyst: Application to Asymmetric Mukaiyama Aldol Reactions of *α*-Ketoesters

**DOI:** 10.3390/molecules17089010

**Published:** 2012-07-30

**Authors:** Ryo Gotoh, Masahiro Yamanaka

**Affiliations:** Department of Chemistry and Research Center for Smart Molecules, Rikkyo University, Toshima-Ku, Tokyo 171-8501, Japan

**Keywords:** Lewis-Brønstedcombined acid, bisamidine, asymmetric Mukaiyama aldol reaction, *α*-ketoester

## Abstract

Focusing on the steric and electronic properties of the resonance-stabilized amidine framework, a cationic metal-bisamidine complex was designed as a conjugated combined Lewis-Brønsted acid catalyst. The chiral Zn(II)-bisamidine catalyst prepared from the 2,2'-bipyridyl derived bisamidine ligand, ZnCl_2_, and AgSbF_6_ promoted asymmetric Mukaiyama aldol reaction of *α*-ketoester and *α*,*α*-disubstituted silyl enol ether to afford the *α*-hydroxyester having sequential quarternary carbons in good yield, albeit with low enantioselectivity. Addition of 1.0 equivalent of the fluoroalcohol having suitable acidity and bulkiness dramatically increased the enantioselectivity (up to 68% *ee*). DFT calculations suggested that this additive effect would be caused by self-assembly of the fluoroalcohol on the Zn(II)-bisamidine catalyst.

## 1. Introduction

**Figure 1:**
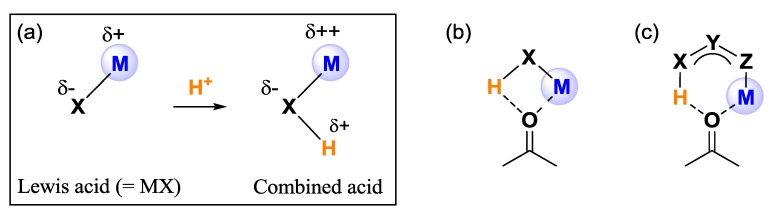
(**a**) Schematic concept of Lewis-Brønstedcombined acid catalyst; (**b**) Double activation by the normal combined acid; (**c**) Double activation by the conjugated combined acid.

Lewis-Brønsted combined acid catalysts promote higher catalytic activity and stereoselectivity than the individual acid catalysts through the dual effects of enhancement of Lewis acidity and the intramolecular hydrogen-bonding interaction of additional Brønsted acid sites (Figure 1a) [1]. Since the late 1990s, various types of Brønsted acid-assisted chiral Lewis acid catalysts have been developed and are regarded as an efficient catalyst design for asymmetric reactions [2,3,4,5,6,7,8,9,10,11,12,13,14]. Based on Yamamoto’s combined acid concept, we proposed double activation phenomena of carbonyl compounds caused by simultaneous coordination of Lewis acid and Brønsted acid sites of the combined acid catalyst. In contrast to the previously reported combined acid catalysts, in which doubly coordination of both acid sites would form a labile four-membered complex (Figure 1b), the three-centered conjugated scaffold would form a more stable six-membered complex (Figure 1c).

**Figure 2:**
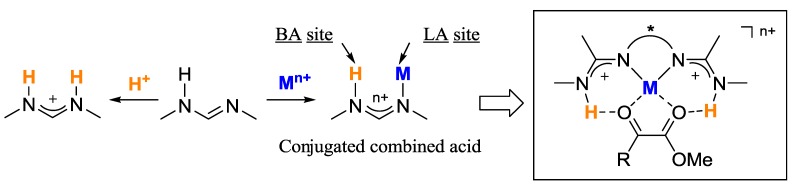
Design of the metal bisamidine complex as a Lewis-Brønstedcombined acid catalyst.

Focusing on the steric and electronic properties of the the resonance-stabilized amidine framework, we designed a novel conjugated Lewis-Brønsted combined acid catalyst. Amidinium cation is significantly stabilized by delocalization of positive charge on two N atoms. The electronic flexibility of the amidine framework should induce similar electronic delocalization in the cationic metal amidine complex. When coordinating of cationic metal ions (M^n+^) instead of proton (H^+^), positive charge in the metal amidine complex could be delocalized to enhance Brønsted acidity of the NH moiety in the parent amidine. We eventually designed the cationic metal bisamidine catalyst, which should be thermodynamically stabilized by bidentate coordination of two amidine moieties (Figure 2).

Various chiral bisamidines have been recently developed as chiral Brønsted acid catalysts [15,16,17,18,19] and chiral ligands [20]. To our best knowledge, however, there has been no report of bisamidine-based combined acid catalysis. For our initial studies, our designed cationic Zn(II)-bisamidine catalyst was applied in the reaction of an *α*-ketoester and an *α*,*α*-disubstituted silyl enol ether to overcome the present limitations of the asymmetric Mukaiyama aldol reaction [21]. The catalytic asymmetric Mukaiyama aldol reaction of *α*-ketoesters has been recently identified as an efficient synthetic tool for making a chiral quaternary carbon center. Evans has reported pioneering studies of asymmetric addition of thioester-derived enolsilanes to pyruvate esters catalyzed by the chiral Cu(II)-bisoxazoline complexes [22,23,24]. Bolm has also reported highly enantioselective additions of various ketone-derived enolsilanes to methyl pyruvate catalyzed by a chiral Cu(II)-oxazolinyl sulfoximine [25,26]. However, the use of aryl substituted *α*-ketoesters or ester-derived enolsilanes is still rather limited. Hoveyda developed the chiral AgF_2_-pyridyl Schiff base catalyzed reaction of alkyl/aryl substituted *α*-ketoesters [27]. Pagenkopf reported a significant expansion in substrate scope to include ester-derived enolsilanes and alkyl/aryl substituted *α*-ketoesters [28]. Despite the synthetic value of *α*-hydroxyesters having sequential quarternary carbons, the reactions of *α*-ketoesters and *α*,*α*-disubstituted silyl enol ethers have been quite rare [29,30].

## 2. Results and Discussion

**Figure 3:**
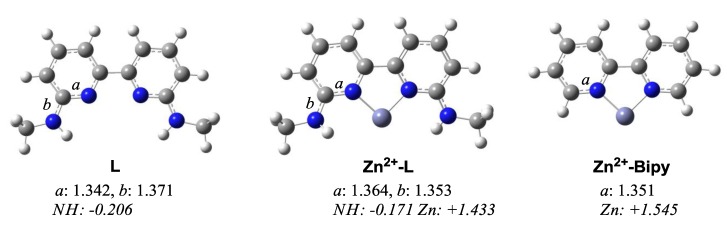
DFT calculations of bisamidine ligand (L), Zn(II)-bisamidine (**Zn^2+^**-**L**), and Zn(II)-bipyridine (**Zn^2+^**-**Bipy**)complexes (B3LYP/LANL2DZ for Zn, 6-31G* for the rest). Bond lengths are shown in Å. NBO charges are shown in *italics*.

As a preliminary computational study, we investigated the dual effects of Lewis and Brønsted acids on the cationic metal bisamidine complex by DFT calculations (Figure 3) [31]. In spite of the structurally stable aromatic ring in the 2,2'-bipyridine framework, the difference of the bond lengths between *a* and *b* in the amidine moiety becomes smaller upon coordination of the Zn(II) cation. The natural charge of the NH moiety in **Zn^2+^**-**L** (−0.171) was more positive than in **L** (−0.206). Furthermore, **Zn^2+^**-**Bipy** exhibited a greater degree of the positive charge on the Zn(II) center (+1.545) than **Zn^2+^**-**L** (+1.433). These natural charge differences suggested delocalization of the positive charge via the amidine framework to increase Brønsted acidity of the NH proton. In a manner similar to the amidinium complex, the efficient charge delocalization in the amidine moiety was also induced in **Zn^2+^**-**L**. These DFT calculations predicted that the cationic Zn(II)-bisamidine complex such as **Zn^2+^**-**L** would have a unique catalytic ability incorporating Lewis acid and Brønsted acid functions. 

First, we compared the catalytic activities of the cationic metal complex prepared with **L1** and **L2**, ZnCl_2_, and 2.0 equivalents of AgSbF_6_ under stoichiometric conditions (Table 1). In the Zn(II), Cu(II), and Fe(II)-catalyzed Mukaiyama aldol reaction of methyl benzoylformate (**1a**) and dimethylketene methyl trimethylsilyl acetal (**2**), **L1** derived catalysts gave the product **3a** in better yields than the corresponding **L2** derived catalyst. This indicates that the presence of the amidine unit is essential for the higher catalytic activity, which would be caused by the Brønsted acid function at the NH moiety. Based on the promising results of the Zn(II)-**L1** catalyst, various counteranions, SbF_6_^−^, BF_4_^−^, and TfO^−^ were explored under catalytic conditions (the reaction was carried out in CH_2_Cl_2_ at −78 °C for 15 h in the presence of 10 mol% Zn(II)-**L1**). The highest yield of 89% was achieved by use of SbF_6_^−^ (BF_4_^−^: 68%, TfO^−^: 57%). 

**Table 1:**
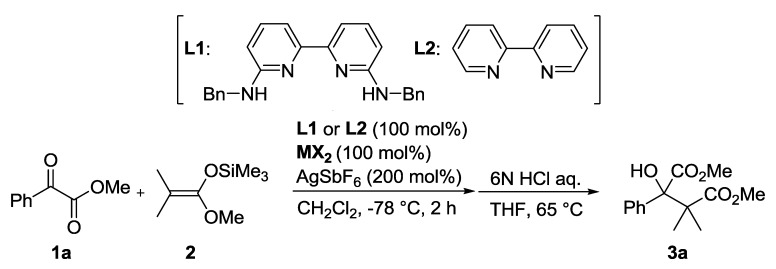
Metal-bisamidine catalyzed reaction of **1a** and **2** under stoichiometric condition.

Entry	MX_2_	Ligand	Yield (%) *^a^*	Ligand	Yield (%) *^a^*
1	CuCl_2_	**L1**	28	**L2**	13
2	ZnCl_2_	**L1**	51 (89) *^b^*	**L2**	30
3	FeCl_2_	**L1**	48	**L2**	15

*Notes*: *^a^* Determined by ^1^H-NMR analysis of crude mixture; *^b^* 10 mol% of Zn(II)-**L1** catalyst was used for 15 h.

**Figure 4:**
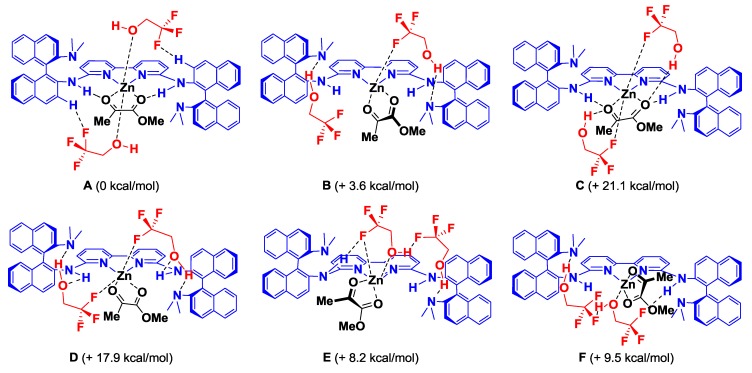
Schematic geometries for the assembled complex models (**A**–**F**) of Zn(II)-**L3** and *α*-ketoester with TFE and the relative energies (B3LYP/LANL2DAZ for Zn, 6-31G* for the rest).

**Figure 5:**
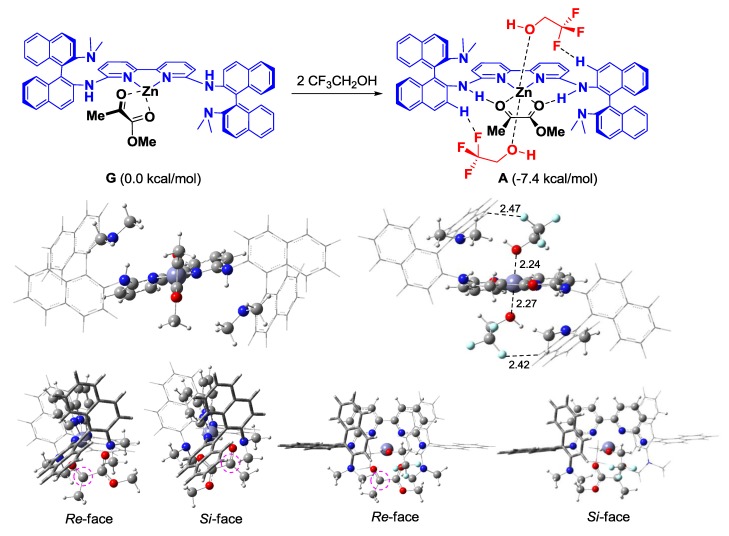
DFT calculations for the TFE addition effect in the complex of Zn(II)-**L3** and *α*-ketoester (B3LYP/LANL2DAZ for Zn, 6-31G* for the rest). Bond lengths are shown in Å.

The (*R*)-DABN derived chiral bisamidine ligand **L3** was developed for the catalytic asymmetric version (Table 2). The asymmetric Mukaiyama aldol reaction of **1a** and **2** was catalyzed by 10 mol% of Zn(II)-**L3** to afford **3a** in moderate yield with 26% *ee* (Entry 1). On the other hand, both Zn(II)-**L4** and Zn(II)-**L5** catalysts proved much less selective (Entries 2 and 3). The NMe_2_ group in the DABN moiety performed better than either smaller or larger amino groups. In some cases, it has been recognized that achiral additives can be beneficial for catalytic activity and the stereocontrol ability of chiral catalysts [32]. Fluoroalcohol, in particular, has emerged as an efficient additive for promoting catalyst turnover in addition reactions of enolsilanes [26,33,34,35]. These reports encouraged us to examine the additive effect of trifluoroethanol (TFE), which acts as a proton source to regenerate the active catalyst or coordinates on the active catalyst to form a more efficient chiral environment at the reaction centre. Addition of 20 mol% of TFE slightly improved yield (71%) and enantioselectivity (35% *ee*, Entry 4). The enantioselectivity was increased with increasing of the amount of the additional TFE. Addition of 1.0 equivalent of TFE provided the optimal enantoselectivity of up to 50% *ee* (Entry 5), while an excess amount of TFE would affect solvent polarity to decrease the enantioselectivity (Entry 6). We predicted that the present additive effect would be caused by self-assembly of the fluoroalcohol on the Zn(II)-**L3** catalyst. To investigate our prediction, DFT calculations for the reactive complex models of the Zn(II)-**L3** and *α*-ketoester with/without TFE were carried out (Figure 4 and Figure 5).

**Scheme 1:**
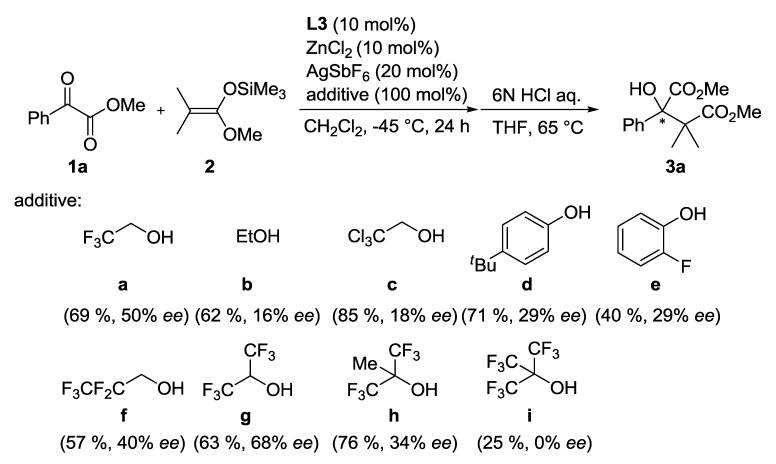
Screening of alcohol additives.

Focusing on the TFE coordination to Zn(II)-**L3** and *α*-ketoester, methyl pyruvate was used in the chemical models to reduce the computational costs. Six assembled complex models (**A**–**F**) were optimized (B3LYP/LANL2DAZ for Zn, 6-31G* for the rest). The assembled complex **A** found to be most stable (Figure 4). The structural properties of the parent complex **G** were dramatically changed by coordination with two molecules of TFE (Figure 5). Whereas **G** has the tetrahedral Zn(II) centre, an octahedral hexacoordinated Zn(II) centre was formed in **A**. The hydrogen-bonding between F and binaphthyl hydrogen atoms (2.42 Å, 2.47 Å) and the Zn-O interaction (2.24 Å, 2.27 Å) stabilized **A** by 7.4 kcal/mol in Gibbs free energy. These structural changes would enhance enantiofacial differentiation at the carbonyl group (dotted purple circles in Figure 5). There is no enantiofacial differentiation at the carbonyl group in **G**. In contrast, *Si*-facial attack of **2** in **A** would be prevented by the steric hindrance of TFE to increase the enantioselectivity. This computational prediction was supported by the stoichiometric reaction with 2.0 equivalents of TFE yielding **3a** with 46% *ee* (almost the same enantioselectivity under catalytic condition, Entry 5 in Table 2). To further improve the enantioselectivity, other alcohol additives were examined (Scheme 1).

**Table 2:**
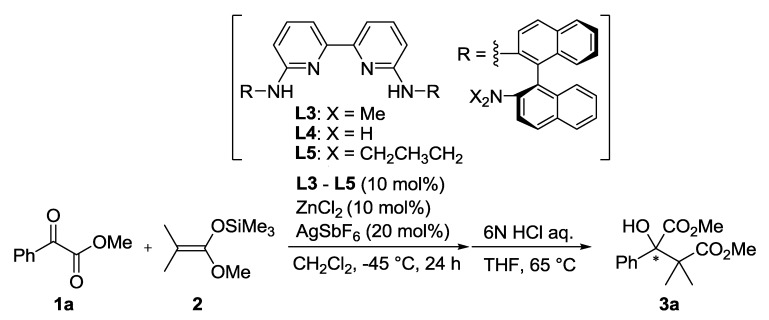
Metal-bisamidine catalyzed reaction of **1a** and **2** under stoichiometric conditions.

Entry	Ligand	Y eq. of TFE	Yield (%) *^a^*	ee (%) *^b^*
1	**L3**	-	60	26
2	**L4**	-	81	1
3	**L5**	-	53	0
4	**L3**	0.2	71	35
5	**L3**	1.0	69	50
6	**L3**	5.0	68	33

*Notes***:***^a^* Determined by ^1^H-NMR analysis of the reaction crude mixtures. *^b^* Determined by chiral HPLC analysis.

**Scheme 2:**
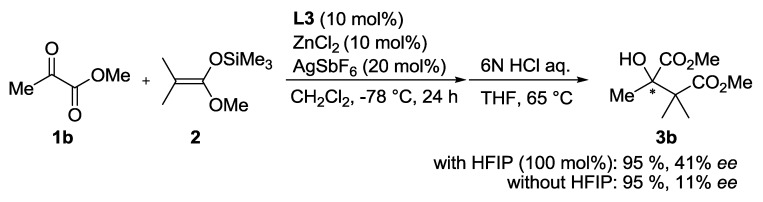
Zn(II)-**L3** catalyzed reaction of **1b** and **2** with or without HFIP.

Whereas ethanol (**b**), trichloroethanol (**c**), and phenol derivatives (**d** and **e**) exhibited no positive impact on the enantioselectivity, 2,2,3,3,3-pentafluoro-1-propanol (**f**) slightly increased the enantioselectivity (40% *ee*). Aliphatic fluorinated alcohols yielded promising results in the initial screening. After investigation of various aliphatic fluorinated alcohols, hexafluoroisopropyl alcohol (HFIP, **g**) was found to be the most efficient alcohol additive and the highest enantioselectivity was achieved (68% *ee*). A more sterically demanding alcohol, 1,1,1,3,3,3-hexafluoro-2-methyl-2-propanol (**h**), exhibited lower enantioselectivity than HFIP (34% *ee*). The more bulky and acidic alcohol tris(trifluoromethyl)methanol (**i**), promoted the achiral pathway to exhibit no enantioselectivity. Therefore, aliphatic fluorinated alcohols having suitable acidity and bulkiness exerted a significant impact on the enantiofacial control of Zn(II)-**L3**. A similar additive effect was observed when methyl pyruvate (**1b**) was used as electrophile. The Zn(II)-**L3** catalyzed reaction of **1b** and **2** readily proceeds at −78 °C to afford **3b** in 95% yield with 11% *ee*. Addition of 1.0 equivalent of HFIP led to the same yield (95%) and an increase in the enantioselectivity to 41% *ee* (Scheme 2). The lower ee value of **3b** than **3a** indicates that Me group would be too small to achieve the enantiofacial discrimination controlled by the Zn(II)-**L3** catalyst.

## 3. Experimental

### 3.1. General

All reactions were performed under nitrogen atmosphere with magnetic stirring in dried glassware. AgOTf, AgBF_4_, ZnCl_2_, CuCl_2_, FeCl_2_·4H_2_O, were purchased from Sigma-Aldrich Japan K. K. (Tokyo, Japan). Dimethylketene methyl trimethylsilyl acetal was purchased from Tokyo Chemical Industry Co., Ltd. (Tokyo, Japan). (*R*)-1,1'-binaphthyl-2,2'-diamine ((*R*)-DABN), methyl phenylglyoxylate, methyl pyruvate were purchased from Wako Pure Chemical Industries, Ltd. (Tokyo, Japan) All dehydrated solvents were purchased from Kanto Chemical Co., Inc. (Tokyo, Japan) Enantiomeric excess was determined on a Shimadzu HPLC system with UV detection or a Shimadzu GC system. ^1^H-NMR and ^13^C-NMR spectra were recorded on a JEOL JNM-ECX400 spectrometer. Chemical shifts in CDCl_3_ were reported in ppm from tetramethylsilane as the internal standard (CDCl_3_: δ = 0) for ^1^H-NMR and from the solvent resonance (CDCl_3_: δ = 77.0) for ^13^C-NMR. Infrared (IR) spectra were recorded on a JASCO FT/IR-230 Fourier transform infrared spectrophotometer. High resolution mass was performed by JEOL JMS-GC mate II. Purification of the products was performed by column chromatography on silica gel 60N (spherical, neutral, Kanto Chemical Co., Inc), Wakogel 50NH_2_ (Wako Pure Chemical Industries, Ltd.) or activated alumina (about 75 μm).

### 3.2. Synthesis of the 2,2'-Bipyridyl Derived Bisamidine Ligands

#### 3.2.1. Synthesis of 6,6'-Dibromo-2,2'-dipyridyl

To a solution of 2,6-dibromopyridine (1.17 g, 5.0 mmol) in Et_2_O (10 mL), *n*-BuLi (1.67 M solution in hexane, 6.6 mL, 11 mmol) was added dropwise at −78 °C over 1 h, and then CuCl_2_ (1.01 g, 7.5 mmol) was added to the reaction mixture. After stirring for 30 min, the reaction mixture was bubbled by O_2_ for 24 h at −78 °C. To the reaction mixture 6 N HCl aq. (15 mL) was added, and then filtered through B üchner funnel. The residue was purified by recrystallization from AcOEt gave 6,6'-dibromo-2,2'-dipyridyl as a white powder in 28% yield.

#### 3.2.2. Synthesis of the (*R*)-DABN Derivatives

(*R*)-*N,N*-Dimethyl-1,1'-binaphthalene-2,2'-diamine [(*R*)-NMe_2_-DABN] was prepared according to the previously reported method [36]. To a solution of (*R*)-1,1'-binaphthalene-2,2'-diamine [(*R*)- DABN] (516.0 mg, 2.0 mmol) and AcOH (1.2 mL, 20 mmol) in CH_2_Cl_2_ (10 mL) was added acetic anhydride (0.2 mL, 2.0 mmol) at 0 °C under N_2_. The reaction mixture was stirred for 20 h at room temperature, and then 1 N NaOH aq. was added to adjust the solution to pH > 7. The solution was extracted by CH_2_Cl_2_ and the combined organic layers were washed with brine, dried over Na_2_SO_4_, and then concentrated under reduced pressure. The crude mixture was purified by column chromatography on silica gel 60N (AcOEt/hexane = 2/1) to afford N-[(*1R*)-2'-amino-1,1'-binaphthalen-2-yl]acetamide [(*R*)-Ac-DABN] as a colorless oil in 75% yield. To a solution of (*R*)-Ac-DABN (0.49 g, 1.5 mmol) and aqueous formaldehyde (37%, 1.48 mL, 17.5 mmol) in THF (19 mL) was added NaBH_3_CN (0.19 mg, 10.3 mmol). The reaction mixture was stirred for 15 min at room temperature followed by addition of AcOH (1.9 mL). After stirring for 4 h at room temperature, 1 N NaOH aq. was added to adjust the solution to pH > 7. The solution was extracted by CH_2_Cl_2_ and the combined organic layers were washed with brine, dried over Na_2_SO_4_, and then concentrated under reduced pressure. The crude mixture was purified by column chromatography on silica gel 60N (AcOEt/hexane = 1/5) to afford the *N*-[(*1R*)-2'-(dimethylamino)-1,1'-binaphthalen-2-yl]acetamide [(*R*)-Ac-NMe_2_-DABN] as a brown powder in quantitative yield. To a solution of (*R*)-Ac-NMe_2_-DABN (529 mg, 1.5 mmol) in EtOH (5 mL) was added 4 N HCl (5 mL). The reaction mixture was stirred for 24 h at 75 °C, and then 1 N NaOH aq. was added to adjust the solution to pH > 7. The solution was extracted by CH_2_Cl_2_ and the combined organic layers were washed with brine, dried over Na_2_SO_4_, and then concentrated under reduced pressure. The crude mixture was purified by column chromatography on silica gel 60N (AcOEt/hexane = 1/10) to afford (*1R*)-*N,N*-dimethyl-1,1'-binaphthalene-2,2'-diamine [(*R*)-NMe_2_-DABN] as a white powder in 90% yield.

To a solution of (*R*)-Ac-DABN (93 mg, 0.29 mmol) in DMF (1.5 mL), 1,5-dibromopentane (345 mg, 1.5 mmol) and Et_3_N (0.3 mL, 3.0 mmol) were added. The reaction mixture was stirred for 2 ddy at 150 °C followed by addition of water (5 mL). The solution was extracted by CH_2_Cl_2_ and the combined organic layers were washed with brine, dried over Na_2_SO_4_, and then concentrated under reduced pressure. To a solution of the crude residue in EtOH (2 mL) was added 6 N HCl aq. (2 mL). After stirring for 24 h at 85 °C, 1 N NaOH aq. was added to adjust the solution to pH > 7. The solution was extracted by CH_2_Cl_2_ and the combined organic layers were washed with brine, dried over Na_2_SO_4_, and then concentrated under reduced pressure. The crude mixture was purified by column chromatography on silica gel 60N (AcOEt/hexane = 1/10) to afford (*1R*)-2'-(1-piperidinyl)-1,1'-binaphthalen-2-amine [(*R*)-C_5_H_10_N-DABN] as a white powder in 66% yield.

#### 3.2.3. Synthesis of **L1**, **L3**, **L4**, and **L5**

2,2'-Bipyridyl linked bisamidines **L1**, **L3**, **L4**, and **L5** were prepared according to the C-N coupling method reported by Buchwald [37]. A solution of Pd(dba)_2_ (6.8 mg, 0.012 mmol) and racemic-BINAP (14.8 mg, 0.024 mmol) in toluene (2 mL) under N_2_ was stirred for 20 min at room temperature. 6,6'-Dibromo-2,2'-dipyridyl (153.5 mg 0.49 mmol), *^t^*BuONa (119.5 mg, 1.25 mmol) and the primary amine (**L1**: BnNH_2_, **L3**: (*R*)-NMe_2_-DABN, **L4**: (*R*)-Ac-DABN, **L5**: (*R*)-C_5_H_10_N-DABN, 1.0 mmol) were added to the solution at room temperature followed by stirring or 24 h at 110 °C. The solution was filtered through Celite and concentrated under reduced pressure. 

For **L1**, **L3**, and **L5**: The crude mixture was purified by column chromatography on activated alumina (**L1**: AcOEt/CH_2_Cl_2_ = 1/1, **L3**: AcOEt/hexane = 1/3) or silica gel 60N (**L5**: AcOEt/hexane = 1/15) to afford the 2,2'-bipyridyl linked bisamidine as a white powder (**L1**: 99%, **L3**: 99%, **L5**: 51%).

For **L4**: To a solution of the crude residue in EtOH (2 mL) was added 6 N HCl aq. (2 mL). After stirring for 24 h at 85 °C, 1 N NaOH aq. was added to adjust the solution to pH > 7. The solution was extracted by CH_2_Cl_2_ and the combined organic layers were washed with brine and dried over Na_2_SO_4_. The crude mixture was purified by column chromatography on silica gel 60N (AcOEt/ hexane = 1/3) to afford **L4** as a white powder in 99% yield.

**L1**: ^1^H-NMR (400 MHz, CDCl_3_): δ 7.64 (d, *J* = 7.6 Hz, 2H), 7.50 (m, 2H) 7.40 (d, *J* = 7.2 Hz, 4H), 7.34 (m, 4H), 7.28 (m, 2H), 6.38 (d, *J* = 7.6 Hz, 2H), 4.97 (br, 2H), 4.60 (s, 4H); ^13^C-NMR (100 MHz, CDCl_3_): δ 158.3, 139.9, 138.5, 128.9, 127.8, 127.8, 127.4, 110.8, 107.2, 46.7; IR ν 3392, 3055, 2831, 2785, 1572, 1500, 1429, 1335 cm^−1^; HRMS Calcd for C_24_H_2__3_N_4_ [M+H]: 367.1923; Found: 367.1948.

**L3**: ^1^H-NMR (400 MHz, CDCl_3_): δ 8.28 (d, *J* = 9.2 Hz 2H), 7.90 (m, 6H), 7.81 (d, *J* = 8.4 Hz, 2H), 7.67 (m, 2H), 7.47 (m, 4H), 7.33(m, 2H), 7.27 (m, 2H), 7.20 (m, 2H), 7.12 (m, 6H), 6.68 (s, 2H), 6.63 (d, *J* = 8.0 Hz, 2H), 2.51 (s, 12H); ^13^C-NMR (100 MHz, CDCl_3_): δ 155.6, 155.0, 150.4, 138.3, 137.2, 134.4, 134.2, 130.5, 130.0, 129.9, 128.4, 128.3, 128.2, 126.8, 126.6, 126.4, 125.6, 124.3, 124.1, 124.0, 122.6, 121.7, 119.6, 112.6, 109.5, 43.6; IR ν 3395, 3054, 2930, 2867, 2831, 2784, 1571, 1501, 1442, 1428, 1334 cm^−1^; HRMS Calcd for C_54_H_45_N_6_ [M+H]: 777.3706; Found: 777.3377.

**L4**: ^1^H-NMR (400 MHz, CDCl_3_): δ 8.57 (d, *J* = 8.8 Hz 2H), 7.98 (d, *J* = 8.8 Hz, 2H) 7.88 (d, *J* = 8.0 Hz, 2H), 7.80 (m, 4H), 7.74 (m, 2H), 7.48 (m, 2H), 7.34 (m, 2H), 7.24–7.04 (m, 12H), 6.53 (d, *J* = 7.6 Hz, 2H), 6.36 (s, 2H), 3.67 (s, 4H); ^13^C-NMR (100 MHz, CDCl_3_): δ 154.8, 154.6, 142.9, 138.2, 137.9, 133.9, 133.3, 130.2, 129.9, 128.7, 128.3, 128.2, 128.1, 127.0, 126.7, 125.0, 124.0, 123.9, 122.5, 120.5, 119.0, 118.2, 113.0, 111.6, 110.5; IR ν 3470, 3381, 3051, 1617, 1463, 1335, 1310, 1270 cm^−1^; HRMS Calcd for C_50_H_37_N_6_ [M+H]: 721.3080; Found: 721.3436.

**L5**: ^1^H-NMR (400 MHz, CDCl_3_): δ 8.30 (d, *J* = 8.8 Hz, 2H), 7.93 (m, 4H) 7.84 (m, 4H), 7.69 (d, *J* = 7.6 Hz, 2H), 7.48 (m, 4H), 7.32 (m, 4H), 7.20–7.05 (m, 8H), 7.00 (d, *J* = 4.0 Hz, 2H), 6.65 (d, *J* = 8.0 Hz, 2H), 2.85 (m, 8H), 1.26–1.17 (m, 12H); ^13^C-NMR (100 MHz, CDCl_3_): δ 155.1, 154.8, 150.4, 138.0, 136.6, 134.1, 133.8, 130.2, 130.1, 129.6, 127.9, 127.9, 126.3, 126.1, 126.1, 125.7, 125.1, 124.0, 123.6, 123.3, 121.1, 121.1, 120.0, 112.1, 109.3, 52.9, 26.1, 24.1; IR ν 3393, 3054, 2929, 2850, 2796, 1572, 1501, 1443, 1429, 1337 cm^−1^; HRMS Calcd for C_60_H_53_N_6_ [M+H]: 857.4332; Found: 857.4039.

### 3.3. General Procedure for Asymmetric Mukaiyama Aldol Reaction of **1a** and **2** (Scheme 1)

Under a N_2_ atmosphere, a solution of ZnCl_2_ (1.4 mg, 0.01 mmol) and **L3** (7.8 mg, 0.01 mmol) in CH_3_CN was stirred at room temperature for 20 min. The solvent was removed under reduced pressure and AgSbF_6_ (6.9 mg, 0.02 mmol) was added under N_2_. The residue was stirred in CH_2_Cl_2_ (0.5 mL) at room temperature for 30 min followed by addition of HFIP (16.8 mg, 0.1 mmol). After stirring at room temperature for 20 min, **1a** (16.4 mg, 0.1 mmol), **2** (17.9 mg, 0.1 mmol) were added to the solution and the mixture was stirred at −45 °C. The reaction was quenched by filtration through silica gel 60N. After concentration under reduced pressure, the residue was dissolved in THF (0.5 mL). 6 N HCl aq. was added and the solution was stirred at 65 °C. The solution was extracted by CH_2_Cl_2_ and the combined organic layers were washed with brine, dried over Na_2_SO_4_, and then concentration under reduced pressure. The crude mixture was purified by column chromatography on silica gel 60N (AcOEt/Hexane = 1/15) to afford **3a** as white crystals (63% yield).

*Dimethyl-2,2-dimethyl-3-hydroxy-3-phenylbutanedioate* (**3a**) [38]. **3a** is a known compound. white crystals: IR ν 3474, 2955, 1706, 1442, 1356 cm^−1^; ^1^H-NMR (400 MHz, CDCl_3_): δ 7.57 (d, *J* = 6.4 Hz, 2H), 7.37–7.30 (m, 3H), 4.70 (s, 1H), 3.81 (s, 3H), 3.74 (s, 3H), 1.36 (s, 3H), 1.13 (s, 3H); ^13^C-NMR (100 MHz, CDCl_3_) δ = 178.7, 174.3, 136.9, 128.0, 127.5, 127.3, 81.9, 52.9, 52.4, 50.4, 22.7, 21.5; EI-MS *m/z* = 266 [M]^+^ for C_14_H_18_O_5_; Enantiomeric excess was determined by chiral HPLC analysis (68% *ee*, DAICEL CHIRALPAK OD-H, 2-propanol/*n*-hexane = 5/95, flow rate 1.0 mL/min, wavelength = 220 nm, t_minor_ = 5.9 min and t_major_ = 7.5 min).

*Dimethyl-3-hydroxy-2,2,3-trimethylbutanedioate* (**3b**) [38]. **3b** is a known compound. pale yellow oil.: IR ν 3511, 2954, 1728, 1439, 1374 cm^−1^; ^1^H-NMR (400 MHz, CDCl_3_): δ 3.94 (s, 1H), 3.77 (s, 3H), 3.70 (s, 3H), 1.43 (s, 3H), 1.28 (s, 6H). ^13^C-NMR (100 MHz, CDCl_3_) δ = 176.8, 175.9, 78.0, 52.7, 52.2, 49.3, 21.6, 20.9, 20.6; EI-MS *m/z* = 205 [M+H]^+^ for C_9_H_16_O_5_; Enantiomeric excess was determined by chiral GC analysis (41% *ee*, Cyclodex-*β* column, 100 °C, t_minor_ = 37.5 min and t_major_ = 38.4 min).

## 4. Conclusions

In summary, we developed asymmetric Mukaiyama aldol reaction of *α*,*α*-disubstituted silyl enol ether and *α*-ketoester promoted by the rationally designed chiral Zn(II)-bisamidine catalyst, which exhibited an unique catalytic ability incorporating Lewis acid and Brønsted acid functions. Aliphatic fluorinated alcohols having suitable acidity and bulkiness dramatically enhanced the enantioselectivity. DFT calculations revealed self-assembly of the fluorinated alcohol exerted a significant impact on the active catalyst structure to enhance enantiofacial control. Although DFT calculations predicted *Re*-facial attack leading to the *R* configured product would be preferred, the absolute configuration of the product has not been determined yet. Studies on further improvement of the cationic metal-bisamidine catalyst system and determination of the absolute configuration of the product are currently underway.

## Supplementary Materials

Supplementary materials can be accessed at: http://www.mdpi.com/1420-3049/17/8//9010/s1.
