# Supplementary material for: Chiral Zn(II)-Bisamidine Complex as a Lewis-Brønsted Combined Acid Catalyst: Application to Asymmetric Mukaiyama Aldol Reactions of α-Ketoesters
Source: Molecules. 2012 Jul 30;17(8):9010–22. doi: 10.3390/molecules17089010 (PMC6268631; doi:10.3390/molecules17089010)
Supplement: Supplementary file 1 [file molecules-17-09010-s001.pdf]

Molecules **2012**, *17*, 9010-9023; doi:10.3390/molecules17089010

OPEN ACCESS

molecules

ISSN 1420-3049

www.mdpi.com/journal/molecules

Article

Chiral Zn(II)-Bisamidine Complex as a Lewis-Brønsted Combined Acid Catalyst: Application to Asymmetric Mukaiyama Aldol Reactions of *α*-Ketoesters

Ryo Gotoh and Masahiro Yamanaka *

Department of Chemistry and Research Center for Smart Molecules, Rikkyo University, Toshima-Ku, Tokyo 171-8501, Japan

* Author to whom correspondence should be addressed; E-Mail: myamanak@rikkyo.ac.jp; Tel./Fax: +81-3-3985-2395.

Received: 11 July 2012; in revised form: 21 July 2012 / Accepted: 21 July 2012 / Published: 30 July 2012

Supporting Information

Cartesian coordinates of stationary points S2

Scanned images of ^{1}H-NMR and ^{13}C-NMR S6

Cartesian coordinates of **A** and **B**

A: SCF Done: E(RB+HF-LYP) = −2859.66791556 a.u.
Sum of electronic and thermal Free Energies = −2858.813119 a.u.

Center Number	Atomic Number	Atomic Type	Coordinates (Angstroms) X	Y	Z
1	1	0	−0.793490	4.319822	−1.396537
2	6	0	−1.314772	3.384689	−1.232854
3	6	0	−2.615822	0.964551	−0.817690
4	6	0	−0.696890	2.331518	−0.579421
5	6	0	−2.616712	3.191747	−1.725830
6	6	0	−3.274055	1.991932	−1.527067
7	1	0	−3.117249	3.998976	−2.251785
8	1	0	−4.293664	1.841549	−1.857610
9	6	0	0.695532	2.395568	−0.062622
10	6	0	3.254500	2.288534	0.986769
11	6	0	1.310407	3.574517	0.326342
12	7	0	1.332084	1.195198	0.068675
13	6	0	2.601316	1.122135	0.536853
14	6	0	2.603265	3.503404	0.872187
15	1	0	0.791166	4.522283	0.254758
16	1	0	3.101126	4.412986	1.194058
17	1	0	4.266555	2.220637	1.364837
18	7	0	3.161491	−0.136480	0.542660
19	1	0	3.143026	−0.633849	−0.363340
20	7	0	−3.186994	−0.246827	−0.503535
21	1	0	−3.104376	−0.534046	0.484366
22	7	0	−1.334795	1.135582	−0.409105
23	30	0	0.009840	−0.350665	−0.011841
24	8	0	−0.099156	−1.852801	1.482292
25	6	0	−0.114567	−3.017123	1.064603
26	6	0	−0.077109	−3.144468	−0.466161
27	8	0	0.027329	−2.116359	−1.152407
28	8	0	−0.165628	−4.357054	−0.917472
29	6	0	−0.176568	−4.574895	−2.367114
30	1	0	0.774215	−4.240286	−2.784271
31	1	0	−0.305919	−5.648223	−2.483252
32	1	0	−1.008001	−4.020315	−2.804220

33	6	0	4.399570	−0.420924	1.219634
34	6	0	5.610500	−0.370247	0.535011
35	6	0	4.319817	−0.838057	2.571044
36	6	0	6.789004	−0.862065	1.203310
37	6	0	5.704996	0.242146	−0.831700
38	6	0	5.450794	−1.236978	3.239919
39	1	0	3.349892	−0.839750	3.060337
40	6	0	6.703183	−1.286409	2.572845
41	6	0	5.081428	−0.318299	−1.953080
42	6	0	6.436257	1.476899	−0.977019
43	1	0	5.394629	−1.547595	4.279653
44	6	0	5.221012	0.297626	−3.225677
45	6	0	6.551342	2.087832	−2.269703
46	6	0	5.939051	1.458137	−3.379190
47	1	0	4.775585	−0.160642	−4.100311
48	1	0	6.044458	1.904311	−4.364572
49	6	0	−4.468143	−0.653487	−1.019694
50	6	0	−5.628895	−0.425428	−0.288572
51	6	0	−4.480799	−1.370785	−2.241333
52	6	0	−6.853107	−1.028651	−0.750989
53	6	0	−5.624194	0.478513	0.909826
54	6	0	−5.655914	−1.888790	−2.727487
55	1	0	−3.545764	−1.500420	−2.779400
56	6	0	−6.861647	−1.756480	−1.989570
57	6	0	−4.936942	0.162456	2.088866
58	6	0	−6.322235	1.736242	0.817093
59	1	0	−5.671787	−2.428509	−3.670514
60	6	0	−4.978215	1.053398	3.194868
61	6	0	−6.340374	2.624502	1.943173
62	6	0	−5.664362	2.240934	3.126116
63	1	0	−4.481318	0.788976	4.120277
64	1	0	−5.695778	2.899885	3.989795
65	7	0	−4.148875	−1.044348	2.163813
66	7	0	4.258878	−1.497489	−1.808269
67	6	0	−4.938163	−2.293639	2.178880
68	1	0	−4.269024	−3.144327	2.008714
69	1	0	−5.440584	−2.436662	3.147489
70	1	0	−5.690610	−2.284962	1.392394
71	6	0	−3.145545	−1.080395	3.232279

72	1	0	−2.482829	−1.934445	3.056613
73	1	0	−2.544176	−0.166782	3.223343
74	1	0	−3.579033	−1.203629	4.236910
75	6	0	5.007444	−2.732541	−1.493770
76	1	0	5.703773	−2.564201	−0.674460
77	1	0	4.301192	−3.514090	−1.192295
78	1	0	5.570153	−3.092250	−2.368536
79	6	0	3.326592	−1.755352	−2.909718
80	1	0	2.627005	−2.535622	−2.589547
81	1	0	2.755533	−0.853559	−3.147921
82	1	0	3.819078	−2.110683	−3.828368
83	6	0	−7.019839	3.869036	1.854069
84	1	0	−7.030333	4.518358	2.725650
85	6	0	−6.976475	2.171752	−0.373216
86	1	0	−6.980464	1.531116	−1.247296
87	6	0	−7.651138	4.248474	0.691641
88	1	0	−8.171074	5.199804	0.633406
89	6	0	−7.618753	3.390225	−0.432098
90	1	0	−8.113075	3.691313	−1.351345
91	6	0	−8.067402	−0.947978	−0.013088
92	1	0	−8.079998	−0.416365	0.931842
93	6	0	−8.072882	−2.340044	−2.448860
94	1	0	−8.066409	−2.880020	−3.391995
95	6	0	−9.230656	−2.232815	−1.712978
96	1	0	−10.150622	−2.684587	−2.071329
97	6	0	−9.221890	−1.537280	−0.480592
98	1	0	−10.135709	−1.466708	0.101863
99	6	0	7.868798	−1.761472	3.231412
100	1	0	7.790874	−2.070937	4.270271
101	6	0	8.047591	−0.966470	0.547366
102	1	0	8.130662	−0.666732	−0.491289
103	6	0	9.072082	−1.838870	2.567610
104	1	0	9.956402	−2.206456	3.079189
105	6	0	9.156331	−1.445386	1.211234
106	1	0	10.105257	−1.520845	0.688615
107	6	0	7.029061	2.159911	0.125727
108	1	0	6.961231	1.733396	1.119600
109	6	0	7.262142	3.309579	−2.417424
110	1	0	7.345327	3.744319	−3.410110

111	6	0	7.831854	3.931537	−1.330907
112	1	0	8.376339	4.862929	−1.452486
113	6	0	7.703607	3.349487	−0.047937
114	1	0	8.149623	3.842311	0.811315
115	6	0	−0.158405	−4.216974	1.936850
116	1	0	−0.179964	−3.922942	2.986890
117	1	0	−1.035992	−4.829724	1.692045
118	1	0	0.716041	−4.852463	1.741836

B: SCF Done: E(RB+HF-LYP) = −3765.23527528 a.u.
Sum of electronic and thermal Free Energies = −3764.272160 a.u.

Center Number	Atomic Number	Atomic Type	Coordinates (Angstroms) X	Y	Z
1	1	0	0.702286	−1.198463	4.591921
2	6	0	1.245530	−1.202579	3.656735
3	6	0	2.587429	−1.236381	1.236493
4	6	0	0.735962	−0.568837	2.532852
5	6	0	2.466873	−1.882278	3.552457
6	6	0	3.150533	−1.893853	2.357066
7	1	0	2.877812	−2.393905	4.417395
8	1	0	4.103796	−2.395080	2.255809
9	6	0	−0.540931	0.196570	2.606816
10	6	0	−2.961661	1.520012	2.691632
11	6	0	−1.012193	0.671507	3.822263
12	7	0	−1.222983	0.409130	1.440363
13	6	0	−2.434656	1.023061	1.475581
14	6	0	−2.237955	1.351745	3.851194
15	1	0	−0.437759	0.545188	4.729908
16	1	0	−2.619809	1.739615	4.790585
17	1	0	−3.919264	2.023688	2.688698
18	7	0	−3.114163	1.180652	0.295362
19	1	0	−2.770851	0.642673	−0.490616
20	7	0	3.234369	−1.237577	0.025013
21	1	0	2.905529	−0.572748	−0.667077
22	7	0	1.378924	−0.624695	1.325684
23	30	0	0.061252	0.000725	−0.191727
24	8	0	1.190021	−0.432016	−2.111923

25	6	0	0.599648	−0.209851	−3.163002
26	6	0	−0.784402	0.454351	−2.985846
27	8	0	−1.237502	0.578850	−1.848205
28	8	0	−1.352411	0.843916	−4.084904
29	6	0	−2.622297	1.574338	−3.987804
30	1	0	−2.400621	2.573912	−3.612890
31	1	0	−2.999465	1.609651	−5.007280
32	1	0	−3.298174	1.042852	−3.316467
33	6	0	−4.527408	1.472248	0.251518
34	6	0	−5.453087	0.434738	0.277685
35	6	0	−4.926061	2.828970	0.179617
36	6	0	−6.851442	0.763715	0.278438
37	6	0	−4.962108	−0.982037	0.383410
38	6	0	−6.261294	3.154970	0.167612
39	1	0	−4.160621	3.598547	0.136265
40	6	0	−7.253696	2.141600	0.224098
41	6	0	−4.449339	−1.679626	−0.720993
42	6	0	−4.935919	−1.587950	1.688759
43	1	0	−6.571905	4.194913	0.116799
44	6	0	−3.842146	−2.956317	−0.505172
45	6	0	−4.318859	−2.869476	1.878131
46	6	0	−3.764719	−3.520055	0.750680
47	1	0	−3.493470	−3.533591	−1.351275
48	1	0	−3.325384	−4.507934	0.872134
49	6	0	4.651769	−1.509626	−0.060012
50	6	0	5.565240	−0.473726	0.092949
51	6	0	5.064278	−2.844594	−0.286872
52	6	0	6.967806	−0.783076	0.043795
53	6	0	5.066999	0.920684	0.346330
54	6	0	6.402883	−3.152865	−0.333243
55	1	0	4.308507	−3.613066	−0.418984
56	6	0	7.384872	−2.141479	−0.165912
57	6	0	4.502586	1.697056	−0.679016
58	6	0	5.096587	1.424135	1.693045
59	1	0	6.724204	−4.176745	−0.503107
60	6	0	3.922052	2.961494	−0.346020
61	6	0	4.504573	2.695331	2.001601
62	6	0	3.915932	3.435242	0.949745
63	1	0	3.548550	3.603043	−1.134556

64	1	0	3.501511	4.418531	1.162548
65	6	0	5.719943	0.943249	−2.695483
66	1	0	5.526917	0.333869	−3.583938
67	1	0	6.218799	1.872259	−3.013060
68	1	0	6.394743	0.393112	−2.047638
69	6	0	3.575504	1.971092	−2.939152
70	1	0	3.488747	1.392701	−3.864553
71	1	0	2.576370	2.106516	−2.518010
72	1	0	3.971902	2.962846	−3.213314
73	6	0	−5.833290	−0.901796	−2.600103
74	1	0	−6.454695	−0.340836	−1.908650
75	1	0	−5.741241	−0.320920	−3.523366
76	1	0	−6.338608	−1.850602	−2.839065
77	6	0	−3.672027	−1.825949	−3.045084
78	1	0	−3.669752	−1.227703	−3.961677
79	1	0	−2.640833	−1.940154	−2.702747
80	1	0	−4.058627	−2.822208	−3.315238
81	6	0	4.532142	3.189941	3.333787
82	1	0	4.092163	4.163100	3.537427
83	6	0	5.683035	0.695893	2.769837
84	1	0	6.145442	−0.264079	2.572836
85	6	0	5.120465	2.463463	4.342075
86	1	0	5.152256	2.852912	5.355049
87	6	0	5.697801	1.203845	4.050290
88	1	0	6.169352	0.634852	4.846571
89	6	0	7.971319	0.215352	0.192455
90	1	0	7.673087	1.247063	0.346848
91	6	0	8.772543	−2.444986	−0.207256
92	1	0	9.076237	−3.476347	−0.365307
93	6	0	9.715608	−1.454794	−0.053282
94	1	0	10.773324	−1.697565	−0.086390
95	6	0	9.308359	−0.113635	0.146113
96	1	0	10.058787	0.662476	0.263867
97	6	0	−8.638163	2.462688	0.231018
98	1	0	−8.931310	3.508293	0.188973
99	6	0	−7.865618	−0.233547	0.340334
100	1	0	−7.577186	−1.278650	0.387459
101	6	0	−9.591484	1.472381	0.292322
102	1	0	−10.646372	1.729183	0.299282

103	6	0	−9.198871	0.113149	0.348148
104	1	0	−9.957445	−0.662263	0.399258
105	6	0	−5.498018	−0.955135	2.836327
106	1	0	−5.984404	0.007020	2.727803
107	6	0	−4.290657	−3.463681	3.168717
108	1	0	−3.830042	−4.442002	3.281551
109	6	0	−4.855303	−2.826610	4.248742
110	1	0	−4.846918	−3.293113	5.229130
111	6	0	−5.463659	−1.560238	4.073526
112	1	0	−5.920302	−1.064223	4.925366
113	7	0	−4.484778	−1.133504	−2.043683
114	7	0	4.438552	1.229657	−2.022766
115	6	0	0.113447	4.091582	−1.523685
116	6	0	0.053296	3.261637	−0.248864
117	6	0	−0.178843	−3.929830	−2.083664
118	6	0	0.127339	−3.248414	−0.756106
119	6	0	1.117635	−0.529450	−4.518268
120	1	0	0.443801	−1.249492	−5.000511
121	1	0	1.112621	0.368225	−5.147776
122	1	0	2.121634	−0.949635	−4.451249
123	9	0	−0.255231	3.353949	−2.605421
124	9	0	1.356110	4.548127	−1.765559
125	9	0	−0.719534	5.132833	−1.427621
126	1	0	0.371509	3.883247	0.592923
127	1	0	−0.979295	2.946270	−0.093403
128	8	0	0.857895	2.085163	−0.357247
129	1	0	1.797783	2.298708	−0.150502
130	8	0	−0.700435	−2.101542	−0.554979
131	1	0	−1.608116	−2.371088	−0.288216
132	1	0	0.007021	−3.978187	0.050239
133	1	0	1.162680	−2.904436	−0.775101
134	9	0	0.734019	−4.872104	−2.335063
135	9	0	−1.398950	−4.500629	−2.080917
136	9	0	−0.162606	−3.034710	−3.107720

Scanned images of ^{1}H-NMR and ^{13}C-NMR

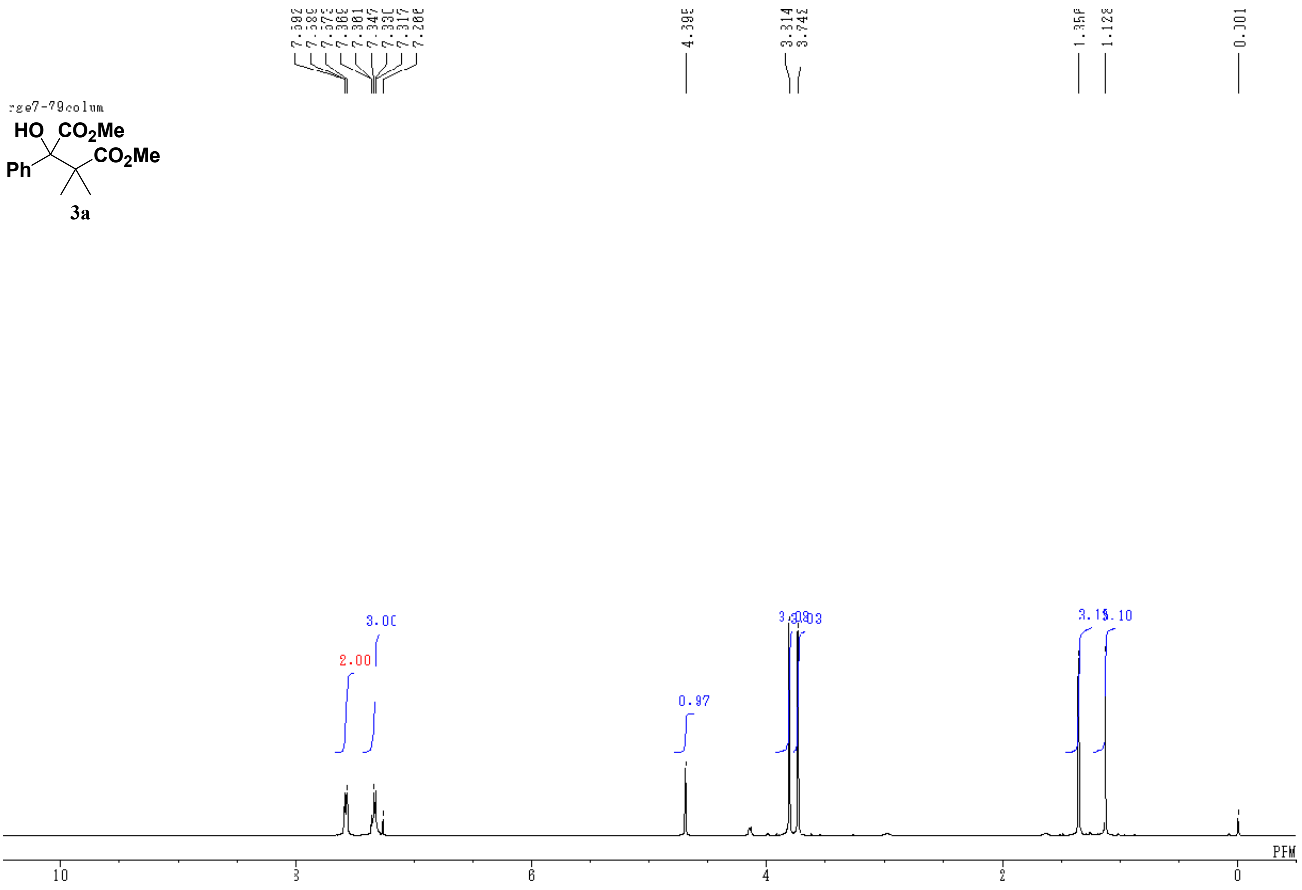

3a

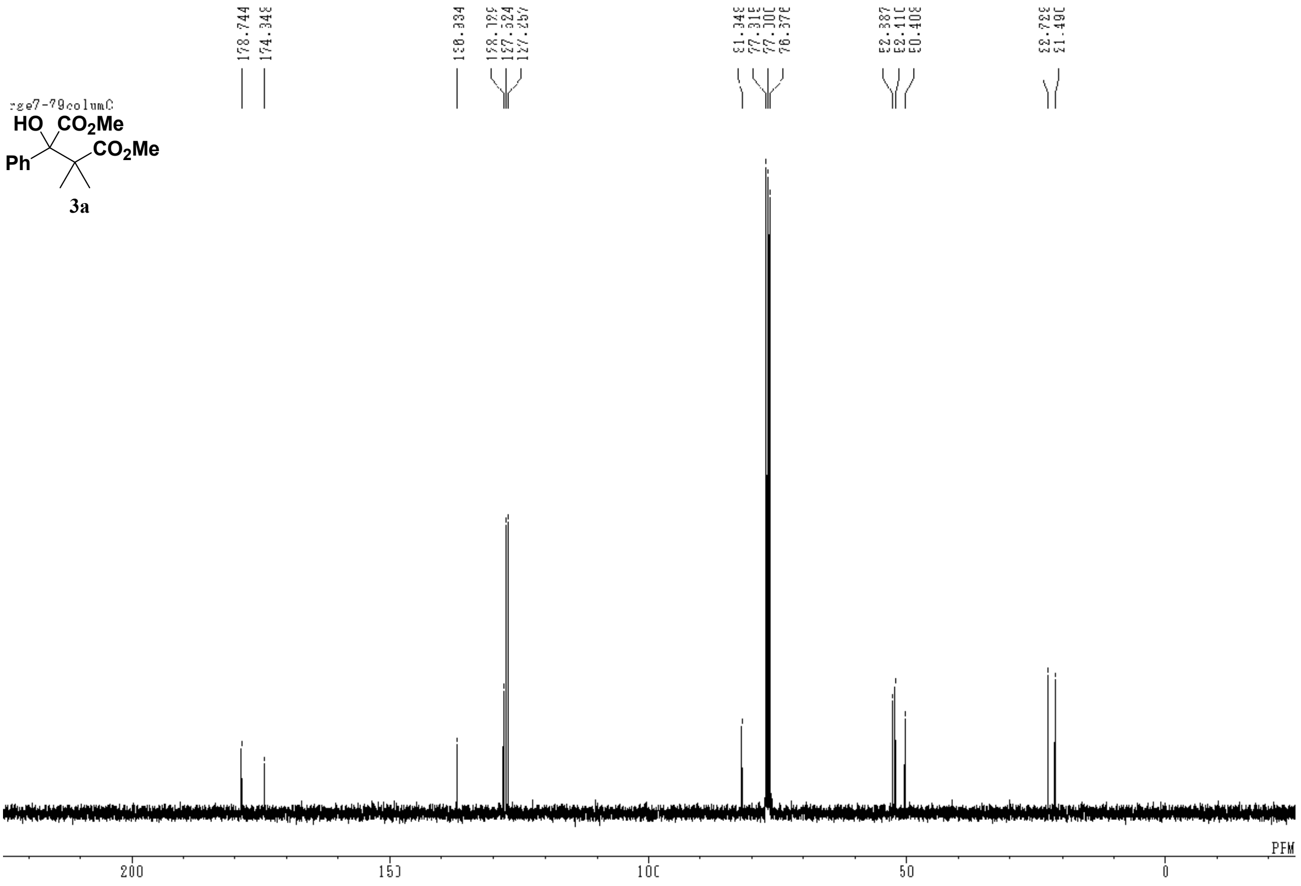

3a

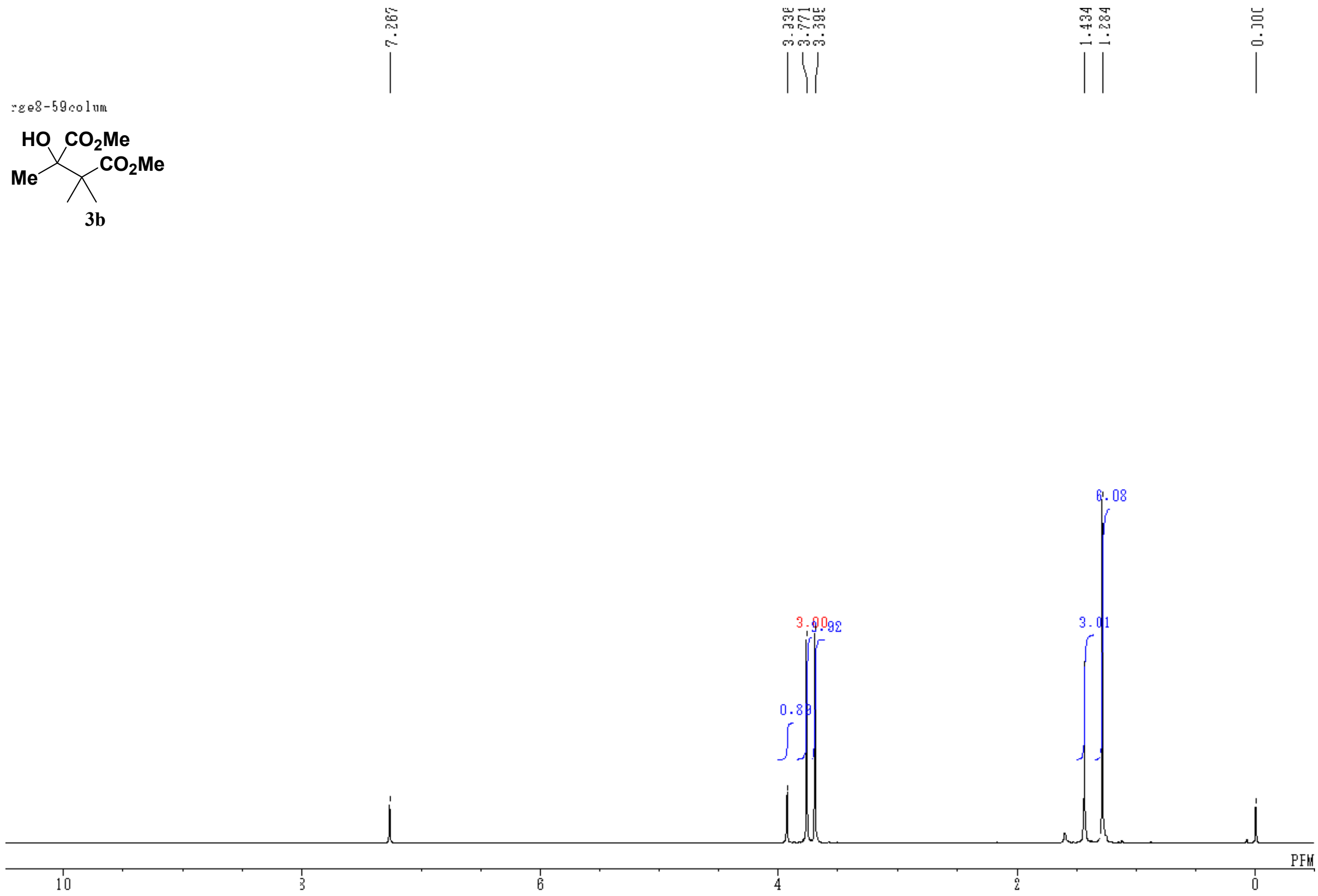
7.267
3.771
1.434
1.284
rge8-59colum
HO CO2Me
CO2Me
Me
3b
0.88
3.00
3.01
PPM
10
4
2
0

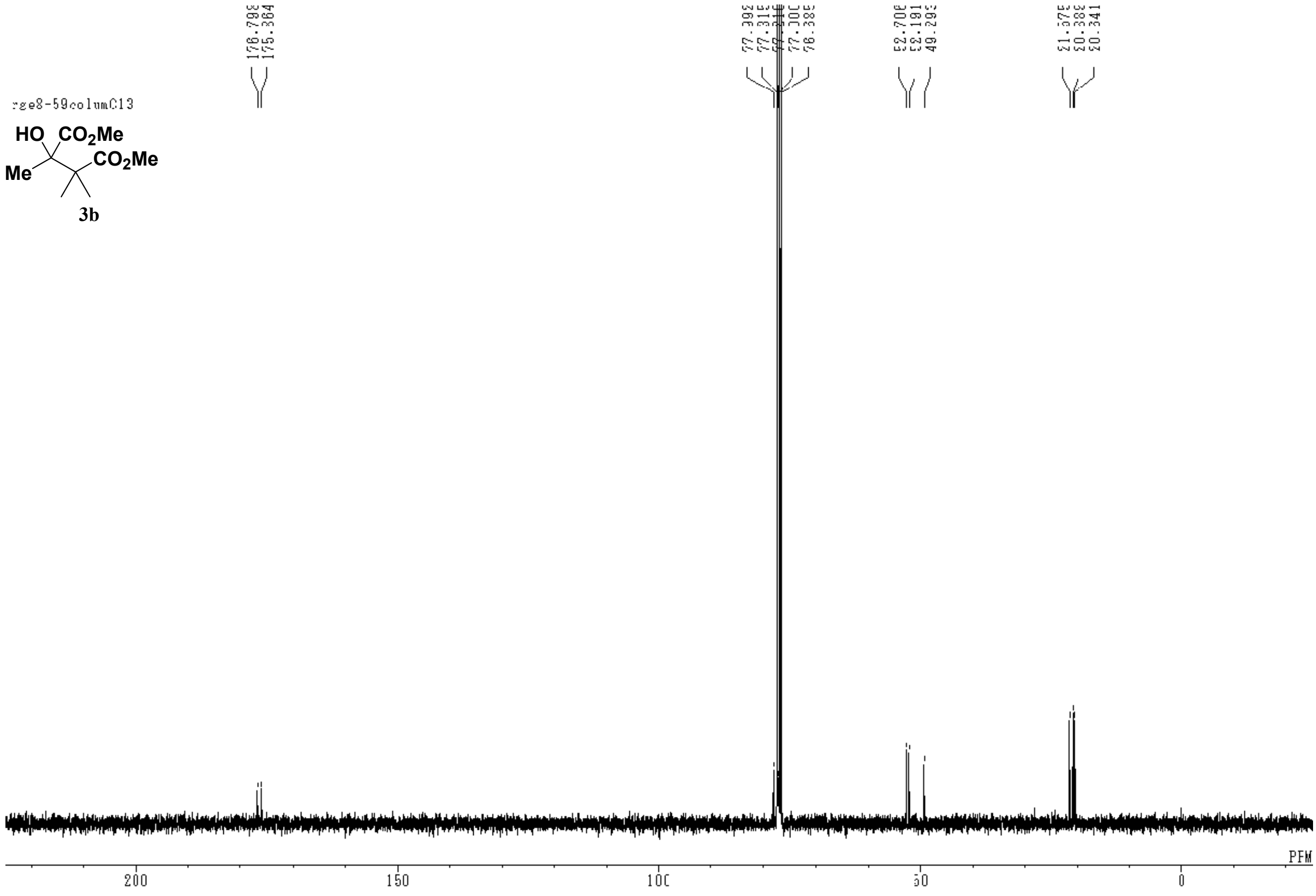
175.384
77.300
49.293
20.341
rge8-59columC13
HO CO2Me
CO2Me
Me
3b
PPM
200
150
0
